# Designing Microparticle-Impregnated Polyelectrolyte Composite: The Combination of ATRP, Fast Azidation, and Click Reaction Using a Single-Catalyst, Single-Pot Strategy

**DOI:** 10.3390/ijms20225582

**Published:** 2019-11-08

**Authors:** Ranjit De, Minhyuk Jung, Hohjai Lee

**Affiliations:** Department of Chemistry, Gwangju Institute of Science and Technology (GIST), Gwangju 61005, Korea; deranjit@gist.ac.kr (R.D.); mhjung817@gist.ac.kr (M.J.)

**Keywords:** composite design, polymer, microparticle, functionalization

## Abstract

Polystyrene microparticles were covalently impregnated into the networks of functional polyelectrolyte chains designed via a tandem run of three reactions: (i) synthesis of water-soluble polyelectrolyte, (ii) fast azidation and (iii) a ‘click’ reaction, using the single-catalyst, single-pot strategy at room temperature in mild aqueous media. The model polyelectrolyte sodium polystyrenesulfonate (NaPSS) was synthesized via the well-controlled atom transfer radical polymerization (ATRP) whose halogen living-end was transformed to azide and subsequently coupled with an alkyne carboxylic acid through a ‘click’ reaction using the same ATRP catalyst, throughout. Halogen to azide transformation was fast and followed the radical pathway, which was explained through a plausible mechanism. Finally, the success of microparticle impregnation into the NaPSS network was evaluated through Kaiser assay and imaging. This versatile synthetic procedure, having a reduced number of discrete reaction steps and eliminated intermediate work-ups, has established a fast and simple pathway to design functional polymers required to fabricate stable polymer-particle composites where the particles are impregnated covalently and controllably.

## 1. Introduction

Biocompatible polymer composites having impregnated nano- and micro-particles are receiving unprecedented attention for the design of advanced materials for drug delivery, regenerative medicine, 3D scaffolds for bone tissue engineering, etc. [1,2,3]. Such particles are surface functionalized by adsorbing, grafting or growing neutral polymers, copolymers, polyelectrolytes, etc., on their surfaces for these purposes [4,5]. Polymer-functionalized microparticles can be packed into desired 3D shaped scaffolds with suitable mechanical stability while the pristine ones cannot [6]. In ultrafiltration, fouling of a membrane can be restricted by altering its net charge via the incorporation of polyelectrolyte-modified particles [7]. Properties like the hydrophilicity/hydrophobicity [8,9], optical behavior [10], mechanical strength [11], conductivity [12], etc., of neat polymer networks can be controlled using particles as fillers. Often, these particles are spread over the polymeric network to design various composite materials. However, it is challenging to distribute such fillers evenly to achieve the anticipated properties in all cases and sometimes they can degrade the material due to their poor bonding with polymer chains [13]. Thus, strong attachment, such as covalent bonding between particles and polymer chains with consequential controlled distribution in the network, is required to overcome this challenge.

To fabricate such soft matter networks, functional polymers with precise architectures are the prerequisite. By taking advantage of these functional groups, particles can be placed precisely at desired locations in the polymer network. To design such polymeric materials, simple procedures having better control over polymerization, minimized numbers of reactions and shortened reaction-durations are anticipated. So far, the most popularly-used method to achieve this is the combination of living polymerization and ‘click’ chemistry [14]. However, the number of discrete reaction steps, the intermediate work-ups and the reaction duration need to be minimized. Recently, Graaf et al. have demonstrated that atom transfer radical polymerization (ATRP), azide group substitution and ‘click’ reaction can be used to synthesize such polymers using the same ATRP catalyst [15].

**Scheme 1:**
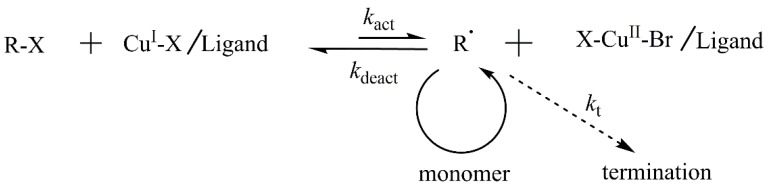
A generalized schematic presentation of the mechanistic pathway involved in a typical ATRP process. Here, R–X is an alkyl halide, and X represents a halogen, pseudohalogen or counterion of the metal ion, and the ligand is bipyridine. Abbreviations. *k*_act_ = rate activation; *k*_deact_ = rate deactivation; *k*_p_ = rate of propagation; *k*_t_ = rate of termination.

ATRP has exhibited good tolerance to functional groups and an extremely small level of bimolecular termination (< a few mol%), allowing the design of polymers with various functionalities, topologies and compositions with enhanced precision [16]. Here, a halide radical (X^•^) generated through the homolytic fission of R–X, transfers to the catalyst Cu^I^/ligand-complex oxidizing it to Cu^II^/ligand-complex (Scheme 1). The other radical species (R^•^) reacts with a monomer possessing an unsaturated group to form an adduct which then further reacts with another monomer to propagate. The dormant chain end-group(s) obtained through ATRP can also be re-activated to resume further propagation and/or transformation to other functional groups via azidation.

The azidation step is mostly carried out using sodium azide (NaN_3_) in organic solvents [17]. Typically, the halogen/pseudohalogen termini of a polymer chain are transformed to azide via nucleophilic substitution (S_N_2) pathway where a large excess of the ionic azide (up to ~10 times) could be required [18]. Reaction duration can also be as long as overnight [19]. Moreover, tertiary halides often require extended reaction durations and elevated temperatures [20]. Besides this, when the halogen group to be substituted is chlorine, it necessitates elevated temperatures due to its stronger bond strength compared to that of C–Br [21]. Additional drawbacks of such S_N_2 pathways are the complex procedures, diminished yields, and more notably, there is a risk of explosion at elevated reaction temperatures in halogenated solvents [22]. Thus, it is necessary to establish an azidation method which is faster, eradicates the need of reaction temperature elevation and can be run in a green solvent using a small amount of the azidating agent. Buxton et al. have shown the oxidation of the transition metal Fe^II^ by azide radical generated from NaN_3_ in aqueous media [23]. Graaf et al. have also demonstrated that sodium azide can be used to azidate a tertiary halide via the radical pathway using Cu^I^ as a catalyst [15]. The reaction occurred much faster than the S_N_2 pathway. About 95% of bromine to azide transformation was reached within 20 min using just 1.2 equivalent of NaN_3_. Once the transformation of halide to azide is done, this can then be used to introduce various functional groups, such as amine, acid, etc.

Popularized by Sharpless et al., the 1,3-dipolar cycloaddition reactions between azides and alkynes have become preeminent among ‘click’ reactions, mainly due to their excellent tolerance towards various functional groups [24]. In polymer science, copper(I)-catalyzed, azide-alkyne cycloaddition (CuAAC) is playing a pivotal role in designing various complex macromolecular architectures and surface engineering [25]. CuAAC can run efficiently at room temperature in aqueous media. It is highly chemoselective, maintains atom economy and occurs rapidly and irreversibly with very low susceptibility to undesired side reactions, ensuring about 100% yields. But, a vital aspect of this reaction is that instability of the catalyst Cu^I^ is often generated in situ using the combination of a Cu^II^ salt and an appropriate reducing agent. Hence, an alternative way could be the use of nitrogen-based ligands, which not only can protect Cu^I^ centers from being oxidized to Cu^II^ but also improve their efficacy [15,26].

Terminal alkynes with different functional groups can, thus, be used to react with azide molecules via the CuAAC reaction to introduce functional groups into target molecules. Graaf et al. have shown the use of such a ‘click’ reaction by incorporating a terminal alkyne-containing fluorophore, dansyl-propargylamide, into an azide end-group containing methacrylate polymer [15]. Thus, the combination of ATRP, azidation and a ‘click’ reaction using a single-catalyst, single-pot strategy has great potential for designing functional macromolecules with precise architectures that can be used for the fabrication of microparticle-impregnated polymer matrixes.

**Scheme 2:**
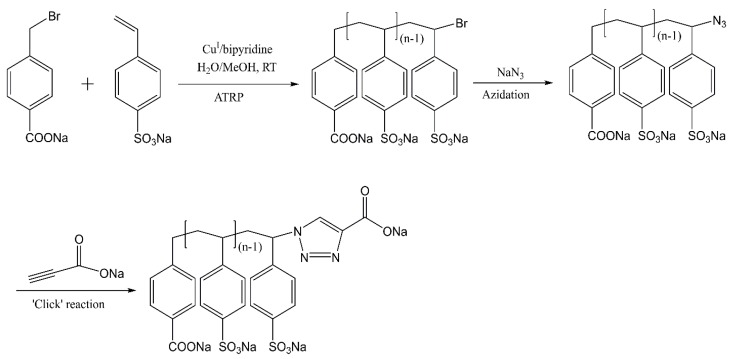
Reaction scheme presenting the three reactions in tandem, ATRP, end-group transformation through azidation and the ‘click’ reaction using single-catalyst, single-pot strategy at room temperature in mild aqueous media (RT: room temperature).

In this work, we report a fast and single-pot strategy to design polymer-microparticle composites having functional polymer sodium polystyrenesulfonate (NaPSS) designed through the tandem approach of three reactions, (i) ATRP, (ii) fast azide group substitution and (iii) ‘click’ chemistry at room temperature in mild aqueous media (Scheme 2). All those three reactions were done in a pot using the same catalyst and without intermediate work-up. Finally, the success of this covalent linking of polystyrene microparticles with polyelectrolyte chains was evaluated through Kaiser assay and imaging.

## 2. Results and Discussion

### 2.1. Homopolymerization of NaSS via ATRP in Water/Methanol Mixed Solvent

**Figure 1:**
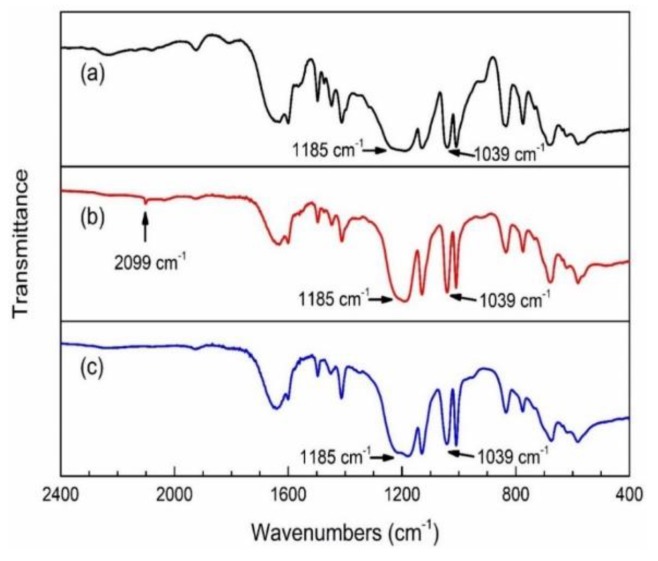
FT-IR spectra of (**a**) NaPSS–Br, (**b**) NaPSS–N_3_ and (**c**) NaPSS–COONa.

Anionic polyelectrolyte NaPSS was synthesized employing typical ATRP condition parameters. The purpose of selecting 4-(bromomethyl)benzoic acid (BMB) as the initiator was to avail the advantage of its acid group to be at a terminal position (say, α-end) of the polyelectrolyte chain. Additionally, its contribution to the back-bone structure and the presence of the benzene ring made it quite similar to the main composition of the NaPSS chain. The presence of bromine in BMB, which is more rapidly substituted due to its comparatively weaker C–Br bond strength than that of C–Cl bond, provided the ease of transforming it into other functional groups. It also contributed better to uphold the living character of the chain-end, which was later transformed to the azide group using free radical mechanism. The use of a 1:1 water-methanol mixture (*v/v*) as the reaction medium can provide better control on the reaction rate than that in pure water or a 3:1 water/methanol mixture (*v/v*) [27]. A decrease in the solvent polarity by the addition of methanol into the water decreases the rate of the propagation [16] Additionally, water is a good ligand for Cu^II^Br_2_ compared to Cu^I^Br; the addition of methanol to water shifts the equilibrium towards the left-hand side, as shown in Scheme 1, providing better control on the overall rate of polymerization. On the other hand, Cu^I^ can also be used along with the optimized amount of Cu^II^ to control the rate of polymerization. Since our purpose was to investigate the use of Cu^I^ for all the three reactions, and the Cu^II^ gets generated in situ through the one-electron transfer during the ATRP reaction, that will be taken advantage of. Hence, all three reactions were carried out without the addition of Cu^II^ from any external source. ATRP is tolerant of the functional groups present in the monomer and that is evident through the FT-IR spectrum (Figure 1a) of the polymer. The peaks at 1185 cm^−1^and 1039 cm^−1^ represent the antisymmetric and symmetric vibrational absorptions of $-{\mathrm{SO}}_{3}^{-}$ group, respectively [28,29].

**Figure 2:**
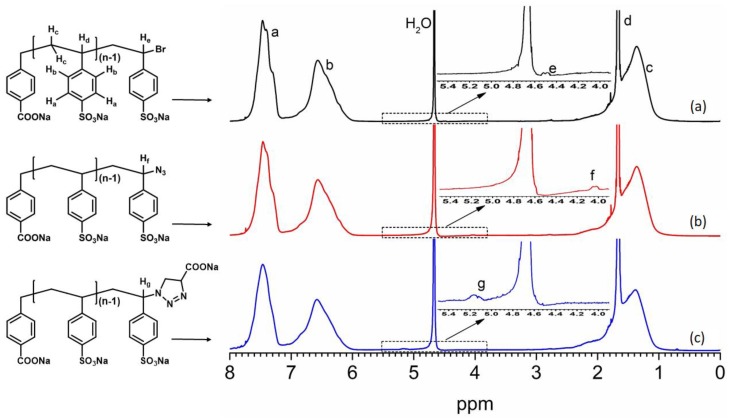
^1^H-NMR (400 MHz) spectra of purified (**a**) NaPSS–Br, (**b**) NaPSS–N_3_ and (**c**) NaPSS–COOH recorded in D_2_O.

The number average molecular weight determined by GPC (Figure S1) was 13,350 g mol^−1^ (polydispersity 1.8) which is close to the value (12,391 g∙mol^−1^) estimated from its ^1^H-NMR spectrum (Figure 2a) following the techniques described in the literature [30]. The integration values of the ^1^H-NMR peaks used for the molecular weight estimation are presented in Table S1, and are followed by detailed calculations. It is notable that the control on the rate of polymerization can further be improved by the use of Cu^I^Cl instead of Cu^I^Br or by the addition of Cu^II^Br_2,_ which was beyond the scope of this work. The polyelectrolyte chain has a –COONa group at the α-end, while there is a Br-group at the ω-end. For simplicity, this chain is hereafter represented as NaPSS–Br. Monomer-to-polymer conversions with the reaction progress were evaluated using ^1^H-NMR spectra of the kinetic samples (data provided in Table S2, presented in Figure S2), which showed that by two hours of reaction duration the conversion reached ~90%. To evaluate monomer conversion, the integration values of the broad signal from 1.07 to 1.89 ppm originated due to the protons of the repeating ethylene group (–CH–CH_2_–) present in the backbone of polymer chain and the signal at 5.28 ppm due to vinylic protons (–CH = CH_2_) of the unconverted monomer were compared (Table S2).

### 2.2. Transformation of NaPSS–Br to NaPSS–N_3_

**Figure 3:**
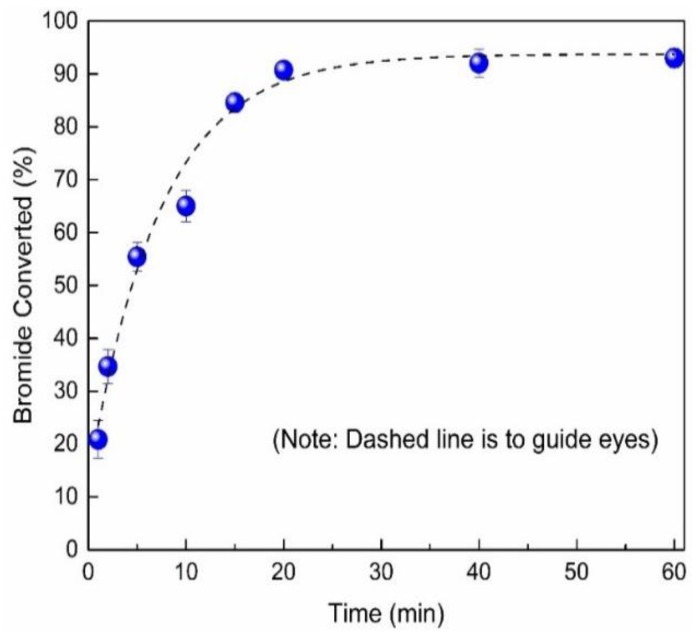
Conversion of bromide to azide as a function of reaction duration in single-catalyst single-pot strategy.

The appearance of a new peak at 2099 cm^−1^ in the FT-IR spectrum (Figure 1b) of NaPSS–N_3_ can be assigned to the stretching vibration of N = N = N which suggests the transformation of bromine to azide [31]. This transformation was also monitored as a function of reaction duration by recording and analyzing ^1^H-NMR spectra of the kinetic samples collected in aliquots during this reaction progress. A new signal was found from 3.99 to 4.09 ppm, which is correlated to methine proton neighboring the azide group (Figure 2b). It was observed that the ratio of integral values of signal from 3.99 to 4.09 ppm to the signal from 4.46 to 4.54 ppm (corresponding to the methine proton neighboring the bromine atom) of the same spectrum gradually increased with the reaction time (Figure S3). After about 40 min of the reaction duration, the signal from 4.46 to 4.54 ppm became very feeble, while the signal from 3.99 to 4.09 ppm rose to prominence. A small upfield shift of the signal from 4.46 to 4.54 ppm to the signal from 3.99 to 4.09 ppm was also observed, which can be attributed to the change in the electronegativity of bromine to nitrogen (Figure 1a,b). The conversions in percentages were calculated and are presented in Figure 3, which shows that after a reaction span of 20 min, about 90% of bromine got converted to azide. Hence, it can be inferred that the bromine to azide conversion took place quantitatively and much faster than the traditional S_N_2 pathway.

Graaf et al. also made a similar observation [15]. The reaction mechanism for this transformation can be established by the following discussion.

**Scheme 3:**
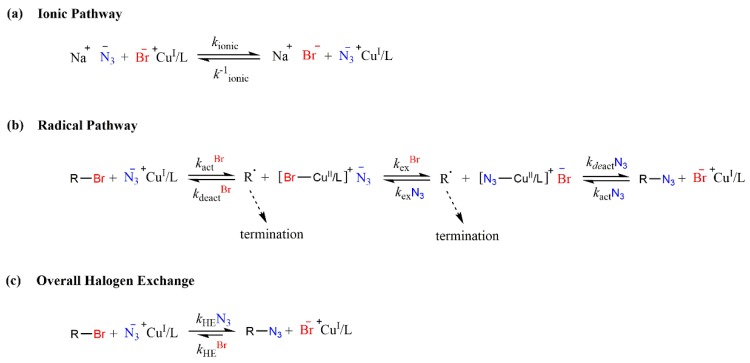
Proposed reaction mechanism for the copper-catalyzed transformation of chain-end group bromide to azide. R–Br: polymer chain with end-group Br, which here can be considered as the macroinitiator; L: ligand; HE: halogen exchange.

It is well-known that in an ATRP process, the transition metal complex (Cu^I^–Y/Ligand) undergoes a ‘single-electron oxidation’ with a concomitant abstraction of halogen or pseudohalogen atom (activation) which comes from an inactive species such as the initiator (R–X). It is then followed by the back-transfer of halogen radical (deactivation) from the metal complex to produce the living chain-end (Scheme 1). Thus, this process continues having both the rates of activation (k_act_) and deactivation (k_deact_). In a well-controlled ATRP, the majority of termination happens through the back transfer of the radical X^•^ or Y^•^ onto the growing chain, which, thereby, continues to possess the living character and can be reactivated later [32]. Such a chain is termed the ‘macroinitiator.’ Matyjaszewski et al. have demonstrated through various model studies that in the use of a ‘mixed halogen’ system, such as R–Br/Cu^I^Cl as the initiator/catalyst pair, ‘halogen exchange’ can occur with the preferential formation of R–Cl over R–Br [33]. The reason is attributed to the stronger C–Cl bond with regard to the C–Br bond. It is also observed that with such a mixed-halide initiator/catalyst system, the majority of the polymer chain-ends can get terminated by chlorine if the initial concentration of Cu^I^Cl is greater than or equal to the initial concentration of the dormant species R-Br (i.e., [Cu–Cl]_0_ ≥ [R–Br]_0_). Thus, it is clear from the above discussion that the catalyst (Cu^I^Y) can incorporate different halogen as well as pseudo-halogen species at the chain-end via the ‘halogen-exchange’ process (Scheme 3b). Here it is important to mention that azides and azide radicals have long been known as pseudohalides due to the resemblance of their chemical behavior to that of halide ions [34]. Bond et al. have shown that the halide anion (Y), which is weakly associated with catalyst complexes Cu^I^(L)Y or X–Cu^II^–Y/Ligand, can undergo ionic reaction [35]. Therefore, once NaN_3_ was added into the reactor where the ATRP reaction was in progress, it could undergo ionic interaction with Cu^I^(L)Y resulting the formation of Cu^I^(L)N_3_ (Scheme 3a) and then proceed with $N_{3}^{•}$ radical generation. It is also known that Cu^II^ can readily oxidize an azide ion [36]. Evans et al. have shown that azide radicals can be realized in a solution phase by electron transfer from dissolved azide [37]. These radicals are one of the most important oxidants used extensively in various one-electron oxidation reactions, such as the one-electron oxidation of phenols, anilines, biological molecules, etc. [38].

Thus, it is clear that the transformation of living end-group bromine to the azide has followed the free radical pathway and a plausible mechanism for this is presented in Scheme 3b. The overall reaction for the halogen exchange (bromide to azide) is summarized in Scheme 3c. The short reaction duration (Figure 3) for the transformation supports this assumption, as was also noticed by Graaf et al. [6]. Once NaN_3_ was added, the preferential azidation over the bromination of polymer chain via the ATRP has taken place, resulting no appreciable change in the molecular weight of the polymer chain. This preferential azidation and unchanged molecular weight can be understood in the light of higher bond dissociation energy of C–N in comparison to that of the C–Br, resulting in a stronger C–N_3_ bond with respect to the C–Br bond [39]. Thus, it is evident that here that the predominant pathway for the transformation of bromine to azide is via the free radical mechanism. The azide radical has, thus, played its role as the radical scavenger in terminating the polymerization reaction in this single-catalyst, single-pot strategy resulting in the formation of NaPSS–N_3_.

### 2.3. Transformation of NaPSS–N_3_ to NaPSS–COONa via ‘Click’ Reaction

The NaPSS-N_3_ obtained was subsequently treated with propiolic acid to carry out the ‘click’ reaction catalyzed by Cu^I^ to obtain the ω-carboxyl-functionalized polystyrenesulfonate chains. The reaction mechanism for this transformation has been presented through Figure S4 prepared following the proposition by Worrell et al. where the dinuclear copper is involved in the transition state [40]. The terminal alkyne propiolic acid is a relatively poor 1,3-dipole acceptor; hence, in the absence of a suitable catalyst the rate of ‘click’ reaction, i.e., the 1,3-dipolar cycloaddition between the alkyne and the dipolar azide—is known to be slow [41]. However, in the presence of Cu^I^, such a reaction can be fast, highly efficient and regioselective [42]. Researchers have often used Cu^II^ sulfate and reduced it in situ to Cu^I^ using a reducing agent, mostly ascorbic acid, particularly for polar reaction media such as water/alcohol mixtures [43]. The other option is to introduce Cu^I^ into the reaction mixture directly [44]. It is also known that Cu^I^ in combination with suitable ligands can enhance the rate of azide-alkyne cyclization significantly [45]. Pyridine derivatives were applied as ligands with notable success to stabilize the copper complex in ‘click’ reactions of macromolecules [46]. Bipyridine-type ligands are excellent candidates to cause effective catalysis in such cyclization processes [47].

Nevertheless, here we have extended the use of the Cu^I^Br/bpy pair as the catalyst/ligand system which was initially used for the synthesis of NaPSS via ATRP. This catalyst/ligand system is an excellent combination for synthesizing the polymer in the first step of the one-pot, one-catalyst strategy, and after that, it was successful in transforming the ω-end bromine group to azide. Hence, this was subsequently extended to ‘click’ reaction in the polar water/methanol (1:1, *v/v*) solvent media which successfully carried out the 1,3-dipolar cycloaddition reaction between azide and alkyne groups. Once this reaction was over, the ^1^H-NMR signal of proton in neighboring methine group to the azide (H_f_, 3.99–4.09 ppm, Figure 2c) disappeared, resulting in the appearance of a new signal from 5.06–5.25 ppm which can be attributed to the methine proton neighboring the triazole ring (H_g_). One more signal was also expected for the proton of the triazole ring in the range of 7 to 8 ppm, but it was not distinguishable due to the presence of strong and broad aromatic proton signals of the polystyrenesulfonate chain. The integration values for both the signals (H_f_ and H_g_) were obtained, and their ratio confirmed the quantitative transformation of the ω-terminal azide group to a carboxyl-triazole end group.

Furthermore, the disappearance of the azide peak from the FT-IR spectrum (Figure 1c) after the ‘click’ reaction suggests the successful execution of the CuAAC reaction [48]. Moreover, the disappearance of this azide peak (2099 cm^−^^1^, Figure 1b) leads us to assume that the density of the triazole was approximately equal to that of the azido group, as the ‘click’ reactions are highly efficient [49]. Thus, it can be inferred that the transformation for azide to the carboxyl-triazole end group was quantitative.

### 2.4. Coupling of Polystyrene Microparticles via the Acid-End-Functionalized Polymer Chains and Kaiser Assay

**Figure 4:**
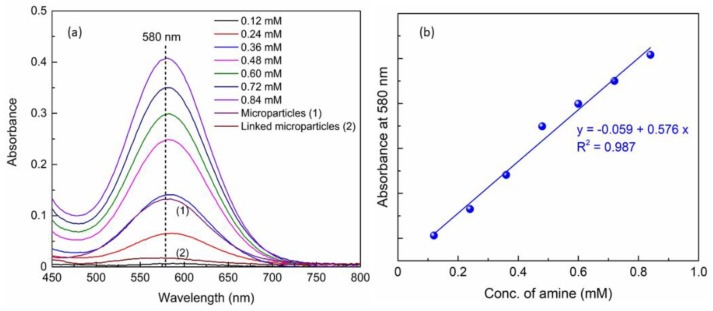
Quantification of amine groups present in hexylamine, as well as pristine and linked microparticles, by Kaiser’s assay. (**a**) Absorption spectra of analyte and (**b**) the corresponding calibration curve prepared using the peak absorbance at 580 ± 2 nm against the concentration of amine in hexylamine solutions ranging from 0.12 mM to 0.84 mM. The amine concentration of both the pristine and linked microparticle samples were within this range.

The functionalized and purified polyelectrolyte (NaOOC–NaPSS–COONa) was then employed to couple polystyrene microparticles by carrying out a high yield amidation between the carboxylate end-groups available at both the chain-ends and the amine groups on microparticle surfaces (Figure S5) to design microparticle impregnated polyelectrolyte network. This amidation was facilitated by 4-(4,6-dimethoxy-1,3,5-triazin-2-yl)-4-methylmorpholinium chloride (DMTMM) in water without controlling the pH as it has a wide working pH range [50]. This is a technique for the one-step amidation of acid and amine groups through covalent bonding for both the small and macromolecules [51]. To evaluate the success of the coupling, quantitative estimation of free primary amine on the surface of the microparticles was carried out using a rapid and sensitive method known as the Kaiser assay, for which the unreacted amines reacted with ninhydrin, and the resulting chromophore called the Ruhemann’s product was determined photometrically using UV–Vis spectroscopy [52]. Ruhemann’s product produced a peak at 580 ± 2 nm (Figure 4a). A calibration curve was realized using the absorption values of this peak for hexylamine solutions of concentrations ranged from 0.12 mM to 0.84 mM (Figure 4b) where absorption peaks for both the pristine and polyelectrolyte-conjugated microparticle samples appeared. The amounts of unreacted amine groups in pristine microparticle and polyelectrolyte-linked microparticle samples were estimated with the help of this calibration curve. This evaluation estimated that more than 60% of amine groups were expended during the amide bond formation.

### 2.5. Imaging of Microparticles Coupled through End-Functionalized Polyelectrolyte (NaCOO–NaPSS–COONa)

**Figure 5:**
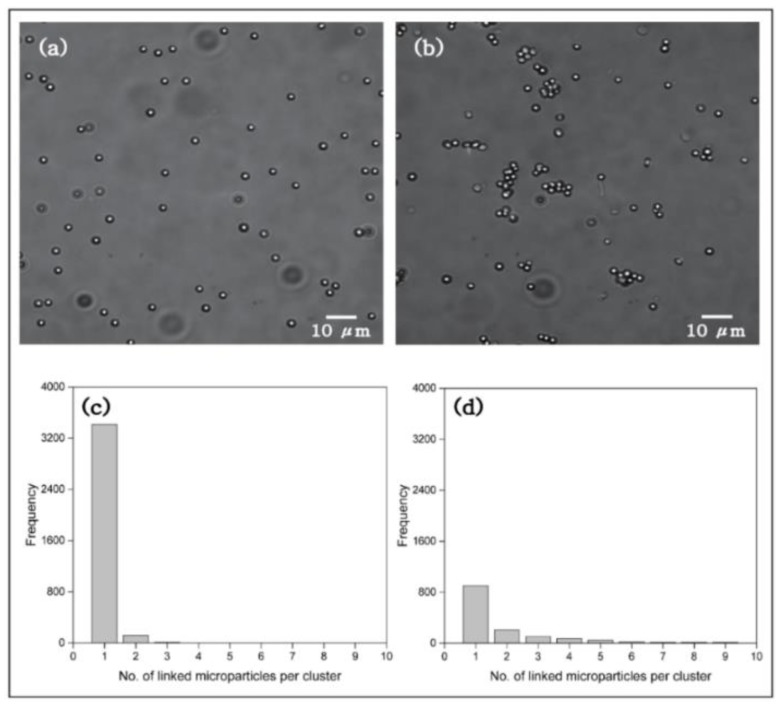
Still images of microparticle samples: (**a**) pristine, (**b**) coupled and (**c**,**d**) their histograms showing the successful coupling by polymer chains designed through ATRP, fast functionalization and ‘click’ chemistry via a single-catalyst, single-pot strategy. Scale bar = 10 µm.

In-plane images of both the pristine and linked microparticles in aqueous dispersion are presented in Figure 5, which demonstrated the success in particle coupling. The image of pristine microparticles shows that the particles were separated from each other (Figure 5a), negating the self-aggregation, while such in-plane images of microparticles coupled through polyelectrolyte chains formed various clusters, each containing a network of several microparticles (Figure 5b). It is worth noting that both the pristine and coupled microparticle samples were sonicated before imaging to avoid any possible, undesired aggregation which would not be caused by coupling through polyelectrolyte chains. To further explore information, histograms were obtained from those images and they are presented in Figure 5c,d for pristine and linked microparticles, respectively. It has been estimated from these histograms, that about 65% of total microparticles were linked through polymer chains. The detailed procedure of this evaluation was carried out using MATLAB program. It is worth mentioning that even though ~60% (as estimated by the Kaiser assay) of the total amine groups present in the aqueous dispersion of microparticles were expended in the amide bond formation with polymer chains, all of these chains could not couple microparticles at both of their ends. Thus, all of the polymer chains were not able to have two microparticles per chain. This resulted in about 65% of total microparticles getting involved in coupling with other microparticles ensuring the formation of those clusters. Considering the average microparticle size as 2.2 µm, the number of pixels per µm as 4.3 and number of pixels per microparticle as 9.5, a thorough evaluation revealed that majority of these clusters were composed of two to five microparticles in each of them. Clusters having six to nine microparticles were also found to exist, though their frequency was low. Among these, clusters consisting of two microparticles were the majority. Clusters having higher than nine microparticles could not be observed. Here, it is worth noting that the imaging could only be conducted on the particles located near the surface of the cover glass. This means that even if clusters of coupled microparticles are comprised of more than nine microparticles, the number of microparticles facing the cover-glass could be less. This is because it is always the two-dimensional face of a three-dimensional object that can be visible through the imaging set up. This can also be understood in the view of the small chain-length of polymer (~50 mers, few tens of nm) in comparison to the large size of microparticles (~2.2 µm). At smaller than the closest approachable distance between microparticles, there is a possibility that particles face steric hindrance due to their large size.

Additionally, even though during the linking process the number of carboxylic groups of the polymer chain used was much higher than that of the amine groups, there is a possibility that all the amine groups of microparticles could not be reached by those polyelectrolyte chains due to their coiling/uncoiling behavior in solution phase [53]. There is also a possibility that after one chain-end got linked to a microparticle, the other end could not due to this coiling/uncoiling behavior. Hence, both the ends of a chain might have not always been available for the coupling with microparticles. To confirm the consistency and reproducibility, a few of the images of linked microparticles from various locations of the sample are presented in Figure S6a–l. In addition, video clips of the flowing aqueous dispersion of microparticles provided in [30]. Details of calculations are also provided in the ESI, have also shown that the pristine microparticles separated from each other (Figure S7a), while the coupled microparticles moved in clusters (Figure S7b). This exploration also supports the observation found through the above-mentioned still images and earlier-discussed Kaiser assay (Section 2.4.).

## 3. Materials and Methods

### 3.1. Materials

Sodium *p*-styrenesulfonate hydrate (TCI, > 93.0%) (NaStS); copper(I) bromide 98% (Sigma-Aldrich, Seoul, South Korea) (Cu^I^Br); 4-(bromomethyl)benzoic acid 97% (Alfa Aesar, Seoul, South Korea) (BMB); 2,2′-bipyridine (bpy) 99.5% (Junsei, Tokyo, Japan); 4-(4,6-dimethoxy-1,3,5-triazin-2-yl)-4-methylmorpholinium chloride (TCI, > 98.0%) (DMTMM); propiolic acid (TCI, > 97.0%) (PA); 1-hexylamine 99% (Sigma-Aldrich) (HA); and ninhydrin (TCI, > 98%) were used as received, without further purification. Silica gel 60 (Merck, 0.063-0.200 mm) was used for the column chromatography while tetrahydrofuran (Duksan, extra pure grade) (THF) was used to improve the purity of the polymer through solvent-precipitation. Deuterium oxide 99.9% D (Cambridge Isotope Laboratories, Inc., Seoul, South Korea) (D_2_O) was used as the NMR solvent. Polystyrene microparticles (5% *w/v*, 2.0–2.2 μm diameter) having surfaces functionalized with amine groups, were procured from Spherotech, IL, Illinois, USA.

### 3.2. Synthesis of Bromine-End-Functionalized Homopolymer NaPSS via ATRP

NaPSS was synthesized via ATRP technique as described elsewhere [27]. Briefly, initiator BMB (0.183 g, 0.85 mmol) was taken in a round-bottomed flask equipped with a magnetic stirrer and having 23 mL of pure water. Sodium hydroxide (1 M) was added dropwise with constant stirring. As the pH rose between 8 to 9, all the acid got dissolved. The volume of water was raised to 25 mL. Monomer NaStS (9.050 g, 38.50 mmol) was added, and the reactor was sealed against the ambient. Stirring continued with continuous purging of argon gas. Earlier argon-purged methanol (25 mL) was added to the reaction mixture, followed by the catalyst Cu^I^Br (0.123 g, 0.85 mmol) and ligand bpy (0.267 g, 1.70 mmol). The colorless reaction mixture turned to dark brown, indicating the initiation of the reaction. The temperature was maintained at 25 °C and the reactor was kept sealed against the ambient. The equivalent monomer:initiator:catalyst:ligand in this polymerization process was maintained as 45:1:1:2. Aliquots of the reaction mixture were taken out at regular intervals using a degassed syringe into an aerated water-methanol mixed solvent. The samples were then freeze-dried, and the monomer to polymer conversion was evaluated from the ^1^H-NMR spectra recorded in D_2_O using a JEOL 400SS, 400 MHz NMR spectrometer (JEOL, Tokyo, Japan), following the procedures described in literature [30]. Details of calculations are also provided in the Electronic Supplementary Information (ESI).

### 3.3. Transformation of Br-End of NaPSS (NaPSS–Br) to Azide (NaPSS-N_3_)

Once the monomer to polymer conversion reached ~90%, as determined by ^1^H-NMR spectroscopy, NaN_3_ (0.066 g, 1.02 mmol which is 1.2 equivalent with respect to the end group of NaPSS–Br) was added to the reaction mixture without any intermediate work-up. FT-IR (Nicolet iS10 FTIR, ThermoFisher Scientific, Waltham, MA, USA) spectra in ATR mode of dried polymer samples before and after the introduction of azide were recorded. To monitor the progress of bromide to azide transformation, aliquots were taken out at regular intervals (1, 2, 5, 10, 15, 20 and 40 min) using a degassed syringe and collected in an aerated water/methanol mixture. After freeze-drying, their ^1^H-NMR spectra were recorded in D_2_O to determine percent conversions of bromide to azide.

### 3.4. Transformation of NaPSS–N_3_ to Acid-End NaPSS (NaPSS–COONa)

After a 1 h reaction duration for the transformation of the end-group from bromide to azide, propiolic acid (53 μL, 0.85 mmol) was injected into the reaction mixture without any intermediate work-up. The color of the reaction mixture continued to dark brown, indicating the equilibrium between Cu^I^ and Cu^II^. Finally, the reaction was terminated by breaking the seal and adding aerated methanol followed by water into it. An aliquot of this product mixture was freeze-dried before purification, and then analyzed using ATR FT-IR and NMR spectroscopy. This diluted solution of NaPSS–COONa was passed through the silica-gel column to separate copper catalyst, and that was followed by the evaporation of the methanol content in a rotary evaporator. This aqueous solution of the polymer product was then precipitated from tetrahydrofuran, filtered and dried. The purity was checked analyzing its ^1^H-NMR spectrum.

### 3.5. Linking of Polystyrene Microparticles Using the NaOOC–NaPSS–COONa Chains

The NaOOC–NaPSS–COONa, synthesized via the single-catalyst single-pot scheme, was then used for linking polystyrene microparticles having surface functionalized by primary amine groups. This linking between carboxylate (–COO^−^) moieties at the polymer chain ends and the amine groups on the microparticle surface was accomplished through amide bond formation facilitated by DMTMM at room temperature in aqueous media [29]. Typically, NaOOC–NaPSS–COOH (21 mg, 1.6 µmol) was taken in a glass vial having water (4.9 mL) equipped with a magnetic stirrer. To which DMTMM (4.4 mg, 16 µmol) and primary-amine functionalized microparticles (100 µL, 5% *w/v*, ~2 × 1016 –NH_2_ groups per µL,) were added and the reaction mixture was continued to stir for 5 h. These polyelectrolyte-coupled microparticles were collected via centrifugation (13,000 rpm for 2 min; Eppendorf Centrifuge 5415 D) and redispersed in distilled water. This sequence of centrifugation followed by redispersion was repeated in triplicate and the final volume was reduced to 1 mL. The solvent of this polymer-coupled microparticle dispersion was exchanged by ethanol and the success of coupling was estimated through a Kaiser assay.

### 3.6. Kaiser Assay

A calibration plot of Ruhemann’s dye was realized to quantify the unreacted primary amine groups on the polyelectrolyte-coupled microparticles using freshly prepared solutions of ninhydrin (0.35%, *w/v*) and HA (1.48 mM) in ethanol. Standard solutions producing final amine concentrations ranging from 0.12 to 84 mM were prepared by combining a constant amount of ninhydrin solution (1 mL) with amine solutions, while ethanol was used as a solvent to adjust the concentrations, leading to the final volume of 5 mL for each sample. The polyelectrolyte-coupled microparticle sample obtained earlier was dispersed to 4 mL of ethanol to which 1 mL of the ninhydrin solution was added. A sample of washed, pristine microparticle dispersion in ethanol was considered as reference. Its amine content was estimated and compared with that of the polyelectrolyte-coupled microparticles. All these samples were heated for 30 min at 60 °C and then cooled down to room temperature, for ~10 min. The two samples having microparticle dispersions were then centrifuged for 5 min at 13,000 rpm using a bench-top centrifuge machine and the clear supernatants were collected. The UV-vis absorption spectra of these two blue supernatant solutions along with the solutions obtained from the standard HA samples were recorded in a UV/visible spectrometer (Ultrospec 2100 pro, Amersham Biosciences, Little Chalfont, UK).

### 3.7. Imaging

**Figure 6:**
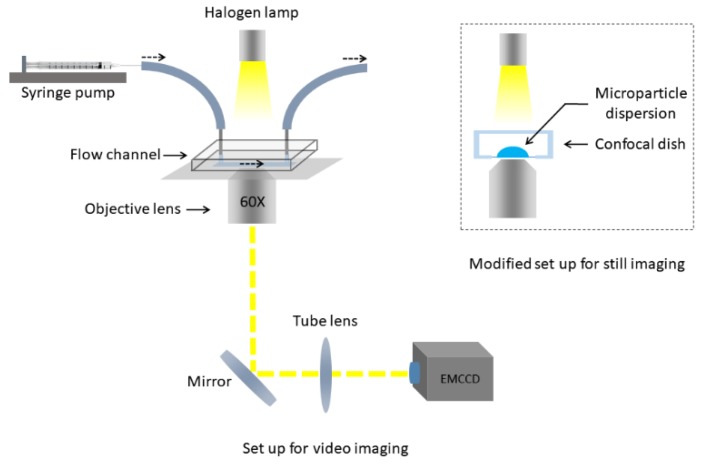
A schematic presentation of the imaging set up used to capture still and video images of the pristine and linked microparticles. The detailed design of the flow-cell is presented in Figure S9.

Both the pristine and linked microparticle dispersion (1 µg/mL) samples were imaged and video-graphed under the same experimental condition parameters using a set up (Figure 6) comprised of a commercial microscope (Olympus, IX73, Tokyo, Japan) fitted with an EMCCD camera (iXon 897, Andor, Belfast, Northern Ireland). To capture still images, a cover glass-bottomed petri dish (SPL Life Sciences) was placed on the stage of the microscope. A total of 300 μL of the aqueous dispersion of pristine or linked microparticles was dropped on the cover glass and was allowed to settle for about 3 h to minimize the movements of microparticles, and thereby to avoid defocusing during imaging. A halogen lamp (U-LH100L-3, Olympus) illuminated the microparticle samples from the top. The light passed through an objective lens (60X, water, UPlanSApo, N.A. 1.20, Olympus) and finally reached the detector of the camera. The microparticles located near the surface of the cover glass were imaged. About 60 in-plane images from different positions of each sample, pristine and linked microparticles, were collected and analyzed to obtain information on the success of coupling, number of particles in each network/cluster, etc. These images were analyzed using home-designed MATLAB codes (Figure S8). To record video clips of the flowing aqueous dispersions of pristine and linked microparticles, the samples were allowed to flow through a home-designed PDMS-cover glass flow-channel controlled by a syringe pump connected to the flow channel through the tubing. Details of the design and preparation of the flow-cell are presented in the ESI (Figure S9). This PDMS channel was placed on the stage and the syringe-pump pushed the syringe containing the microparticle dispersion at a constant rate of 40 μL/min. The videos and images were collected using the EMCCD (20 fps) and rendered through ImageJ (NIH).

## 4. Conclusions

A polyelectrolyte was designed via the combination of ATRP, fast azidation, and ‘click’ chemistry using a single-catalyst, single-pot strategy at room temperature in mild aqueous media via the tandem reaction approach. The anionic polyelectrolyte NaPSS was synthesized via ATRP, whose terminal bromine atom was transformed to azide and subsequently coupled with propiolic acid via 1,3-dipolar cycloaddition reaction using ‘click’ chemistry. It was demonstrated that the bromine to azide transformation followed the free-radical pathway which is faster than the traditional S_N_2 pathway. A plausible reaction mechanism for this step has been provided. All three reactions were carried out using the same Cu^I^ catalyst in one pot without any intermediate work-up. This is a simplified and efficient method that can be extended as a universal technique to design various other functional polymers. Finally, the as-prepared polyelectrolyte chains were used to design composites by controllably linking the polyelectrolyte with polystyrene microparticles. The success of polyelectrolyte–microparticle coupling was established through a Kaiser assay and imaging. About 65% of the microparticles took part in the formation of microparticle-impregnated polyelectrolyte composites. Thus, this versatile method paves a simple, fast and novel approach to prepare various composites comprised of covalently impregnated particles into the polymer networks where they can be placed controllably at the desired location of the network resulting high and evenly distributed mechanical stability. These types of composites can also reduce the degradation of membrane properties, which often occurs during the regeneration of fouled membranes in water treatment technology.

## Supplementary Materials

The following can be found at https://www.mdpi.com/1422-0067/20/22/5582/s1. Figure S1: GPC traces for sodium polystyrene sulfonate (NaPSSBr) synthesized by ATRP using bromomethylbenzoic acid sodium salt and Cu^I^Br as initiator and catalyst (target DP = 45), respectively. The solvent used was 1:1 (*v*/*v*) of water/methanol. Figure S2: Monomer to polymer conversion as a function of reaction duration showing the living nature of the polymerization carried out under single-catalyst single-pot strategy using Cu^I^Br as catalyst and sodium 4-bromomethylbenzoic acid as an initiator at room temperature in 1:1 volumetric ratio of water/methanol; Figure S3: Progress of the conversion from bromide to azide with reaction duration. Information on bromide to azide conversion was obtained by comparing the areas of azide peak (3.99–4.09 ppm) to its corresponding bromide peak (4.46–4.54 ppm) in their 1H-NMR spectrum. Figure S4: Reaction mechanism involving the dinuclear copper intermediate for the copper(I) catalyzed 1,3-cycloaddition between terminal alkyne and azide groups. R-N_3_: polymer chain with end-group N_3_; L: ligand. Figure S5: Use of the end-functionalized polymer chains to conjugate amino-functionalized polystyrene microparticles; Figure S6: Images (a-l) of linked microparticle samples captured from different locations of a sample; Figure S7: Videos of the aqueous dispersion of (a) pristine and (b) linked microparticles flowing through a flow channel. (To watch the videos select the video by a single click on it and then double click on it to play). Figure S8: Analysis of the images of pristine (a–c) and linked (d–f) microparticles through MATLAB coding: (a) Original image of pristine microparticle, (b) black and white version of the same image (a) and (c) inspection of aggregated particles through color-coding; (d–f) are the images obtained following the same procedure. The scale bars represent 10 μm. Figure S9: A schematic presentation of the home-designed diagram of the flow-cell used for video imaging; Table S1: Integration values used for the determination of the molecular weight of bromine end-functionalized sodium polystyrenesulfonate; Table S2: Monomer to polymer conversion with the span of reaction duration.
